# Assessing intake and consumption level of spices among urban and rural households of Ludhiana district of Punjab, India

**DOI:** 10.1186/s12937-020-00639-4

**Published:** 2020-11-06

**Authors:** Simranpreet Kaur Bhathal, Harpreet Kaur, Kiran Bains, Amrit Kaur Mahal

**Affiliations:** 1 Department of Food and Nutrition, Punjab Agricultural University, Ludhiana, Punjab India; 2 Department of Mathematics, Statistics and Physics, Punjab Agricultural University, Ludhiana, Punjab India

**Keywords:** Spices, Consumption, Frequency, Intake, Portion size

## Abstract

**Background:**

Spices are the esoteric food adjuncts that are used for enhancing the sensory quality of the food in Punjabi diets and add many health benefits. Estimating the intake of spices at the individual level is a challenging task as they are consumed in very small quantities as compared to other foods. The present study aimed to assess the intake and spices consumption level of spices among urban and rural households.

**Methods:**

A study was carried out among 100 households each from urban and rural areas from Ludhiana district of Punjab, India to collect the information regarding frequency of spice usage and portion sizes using a questionnaire. The information pertaining to sociodemographic characteristics of female respondents from urban and rural households were also collected. The commonly used 25 spices in Punjabi diets were selected to assess their dietary intake at the individual level among households.

**Results:**

Spice consumption frequency was more in urban households in comparison to rural households. The maximum mean consumption frequency score among urban and rural households was observed for red chilli powder (5.00) and turmeric powder (5.00). Maximum percentage (76 and 72%) of urban and rural households preferred to use the unroasted form of spices, respectively. The highest mean intake and range was observed for red chilli powder (3.19 g with range 0.35–5.23 g) among urban women and (2.41 g with range 0.25–3.75 g) for rural women. Spice intake from individual dishes showed the maximum number of portion sizes for red chilli powder that were from vegetable*>dhal > curry* preparations among urban and *dhal* > vegetable*>curry* preparation among rural households. Average amount of spices consumed by urban adult women was 10.04 g per day which was higher as compared to spices consumed by rural adult women per day (7.68 g).

**Conclusions:**

The study concluded that the urban households showed higher consumption of spices as compared to rural households thus assessing the quantifying intake of spices. Urban adult women consumed more spices per day as compared to rural women. Therefore, more encouragement for increased use of spices is required to reap various health benefits of spices in combating metabolic disorders.

## Background

In the culinary arts, spices are the esoteric food adjuncts that are used for enhancing the sensory quality of the food. The leaf or the herbaceous part of a fresh plant used for flavoring the food preparation is often referred to as a culinary herb where as, any dried part of the plant is known as a spice [1]. Thus, spices can be the bud (cloves), the stigma of flower (saffron), root (dried ginger), aromatic seeds (cumin seeds), berries (peppercorn), bark (cinnamon), etc. Spices have been effectively used in the indigenous systems of medicine in many countries. Due to the redox properties and capacity to block the production of reactive oxygen species, phenolic compounds present in herbs, spices, some fruits and vegetables are being closely associated with their antioxidant activity. Many of the effective antioxidant compounds present that can be derived from spices are of great interest to clinicians as well as biologists due to their ability to protect the human body against various inflammatory processes and oxidative stress [2]. In context to spices which are consumed in very small quantities and are considered as a good source of antioxidants contribute to the total antioxidant activity of the whole meal. Furthermore, being the source of antioxidants, spices also warrant further attention to deal with age-related degenerative disorders and several metabolic diseases [3]. India is an ideal setting to evaluate the consumption of these food items as they are key components of traditional diets. A few small studies in India have assessed these foods but have generally been limited by querying a small number of food items or utilizing methods that may not be practical in large population-based studies [4–6]. Spice intake determination is being acknowledged as they possess bioactive compounds and antioxidant properties. At an individual level, spice intake measurement is a tough and challenging task than estimating the intake of staple foods like cereals and other foods that are consumed in large quantities daily. The world in recent years has witnessed many changes in terms of economy, culture and social life of the people, different demographics, productivity changes, changes in the preferences of domestic consumers for food items, changes in consumption, social factors and globalization. This has led to a change in the food habits of the people, ultimately affecting the spice consumption.

In the present study, an attempt was made to assess the intake and consumption level of spices among adult women of urban and rural households based on the frequency of spice usage and portion size of spice consumed.

## Methods

### Selection of households and spices

The study population consisted of selected 100 urban and 100 rural households in different areas of Ludhiana district of Punjab, India. The households belonged to different socioeconomic groups and to different religions. One day intake was noted from adult women from urban and rural households. The commonly used 25 spices in Punjabi diets, were selected to assess their dietary intake among households. Standard household measuring spoons that measure 1 teaspoon, 1/2 teaspoon and 1/4th teaspoon were used to standardize the weight of each selected whole or powdered form of spices as most of them are used in small quantities. Further, some of the spices used in a minute quantity were verified with an electronic balance.

### Development of questionnaire and data collection

An interview schedule cum questionnaire was developed to elicit the information pertaining to the sociodemographic characteristics, the pattern of spice use and intake, frequency of usage, the quantity of spices in routine dishes in each household. The questionnaire was pretested in ten households and was found satisfactory for collecting the required information. The final questionnaire was prepared by undertaking a pilot study involving suitable urban and rural female respondents to ensure the validity of the questionnaire which was excluded from the final sample. The required data were collected using a pre-structured non-disguised questionnaire cum interview schedule from female respondents in the households, who were involved in a cooking activity to elicit information regarding the spice consumption as described in the questionnaire. An individual if does not cook most of their own food, they may not know all the food items contained in mixed dishes, therefore the food preparer was selected to provide information on items that added during cooking.

### Consumption frequency and spice intake

Spice intake pattern in urban and rural households was assessed based on the spice usage in the households which include the information regarding the type of spice used, intake and frequency of usage with options of ‘daily’, ‘twice’ or ‘thrice’ a week, weekly and occasionally options as described in the questionnaire. For the easy identification of various spices, common names were also mentioned in the questionnaire. Mean consumption frequency scores were also calculated by giving scores: daily (5), thrice a week (4), twice a week (3), weekly (2) and occasionally (1). For the spice intake, the detailed recipe was noted regarding the type and quantity of different spices added to routine dishes. The most frequently used spices in these dishes were identified by the number of households using them. For each meal consumed by responding women herself, name of the dishes and portion size of spices in individual dishes were elicited using a 24-h recall method. To calculate the portion size of the added spice consumed, the quantity of prepared dish consumed by an individual and number of portions obtained from that quantity was noted. One day 24-h recall method determined the quantity of spice intake by an individual based on the spice intake per portion size consumed from all dishes in 1 day. Spice intake per portion size of each individual dish consumed like *dhal*, vegetable and *curry* were also noted separately from adult women of urban and rural households.

### Statistical analysis

The intake of individual spices was presented as frequency intake. The quantity of spices intake was expressed as mean ± standard deviation (S.D), median, 90th percentile and ranges. Statistically, results were analyzed using Chi-square test of independence of attributes. Wilcoxon-Mann-Whitney test was used to compare the mean consumption frequency of spices among the adult women of urban and rural households. Results were considered statistically significant at *p* < 0.01, *p* < 0.05. For the analysis of data SPSS (Statistical Package for the Social Sciences) and SAS (Statistical Analysis System) were used.

## Results

In recent times, consumption frequency and measurement of dietary intake of spices is gaining much significance due to various phytochemicals and presence of antioxidant potential in spices that have been recognized to have health-promoting benefits and protective role against chronic diseases.

### Sociodemographic characteristics of female respondents from urban and rural households

The sociodemographic characteristics of female respondents from urban and rural households is given in Table 1. All the selected respondents were female who performed the cooking activities in the households. The majority of the urban (67%) and rural (56%) respondents belong to 40–60 years age group. It was observed that 53 and 15% of respondents were postgraduate in urban and rural areas, respectively. Further, majority of rural respondents (43%) studied up to the secondary level and only 19% were graduate. While in urban areas, 33% of respondents were graduate, 10% studied up to the secondary level and the remaining 4% studied up to the primary level of education. The majority of rural respondents (57%) were homemakers and urban households (68%) were involved in service. Sixty-five percent of urban respondents belonged to nuclear families whereas, 71% of rural respondents belonged to the joint families. It was observed that 62% of urban respondents were in the category of up to 4 members in the family whereas 53% of rural respondents fall in the category of 4–7 members in a family. Fifty-eight percent of rural respondents reported their monthly family income below Rs 50,000 while 55% of urban respondents had their monthly family income between Rs 50,000 to Rs 1,00,000.

**Table 1:** Sociodemographic characteristics of female respondents from urban and rural households

Particulars	Urban (***n*** = 100)	Rural (***n*** = 100)
**Age (years)**		
25–39	33	44
40–60	67	56
**Education level**		
Primary	4	23
Secondary	10	43
Graduate	33	19
Postgraduate	53	15
**Occupation**		
Service	68	35
Homemaker	19	57
Business	13	8
**Type of family**		
Nuclear	65	29
Joint	35	71
**Family size** (members)		
Upto 4	62	25
4–7	34	53
> 7	4	22
**Family income (Rs/month)**		
> 1,00,000	8	4
50,000-1,00,000	55	38
< 50,000	37	58

### Consumption frequency of spices

The consumption frequency of spices among urban and rural households is shown in Table 2. A spice intake survey among urban households showed that all the urban households consumed red chilli powder and turmeric powder daily. Spices consumed by more than 90% of urban households were cumin seeds (99%), green cardamom (94%), asafoetida, fennel seeds and carom seeds, black pepper (93% each) and coriander seeds (91%). Consumption frequency of dill, saffron, mace, nigella seeds, nutmeg, mace and star anise were found to be lower, less than 50% of urban households consumed these spices. The least consumed spice was star anise as 30% of urban households preferred to consume this on an occasional basis. Contrarily among rural households, red chilli powder and turmeric powder were consumed by all the households and only 13% of households consumed star anise which was least consumed among all 25 spices studied. Among rural households, the top six most frequently consumed spices (higher category) were red chilli powder and turmeric powder consumed on daily basis by households followed by cumin seeds (97%), carom seeds (91%), black pepper (91%), asafoetida (90%). Further, intermediate spice consumption frequency ranged between 89 and 63% of households consuming fennel seeds, coriander seeds, green cardamom, dry mango powder, mustard seeds, fenugreek seeds, pomegranate seeds, black salt, cinnamon and cloves. Spices consumed on a weekly or on occasional basis were gooseberry powder, bay leaf, black cardamom, saffron, nutmeg, mace, nigella seeds, dill and star anise contributing less than 50% of adult women in rural households consuming these spices.

**Table 2:** Consumption frequency of spices among urban and rural households

SPICES	FREQUENCY												
	No. of Households	URBAN (***n*** = 100)					No. of Households	RURAL(***n*** = 100)					
		Daily	Days/Week		Weekly	Occasio-nally		Daily	Days/Week		Weekly	Occasi-onally	***p*** value
			Thrice	Twice					Thrice	Twice			
Red chilli powder	100	100	0	0	0	0	100	100	0	0	0	0	–
Turmeric powder	100	100	0	0	0	0	100	100	0	0	0	0	–
Cumin seeds	99	78	18	1	1	1	97	56	16	8	13	4	0.003
Green cardamom	94	40	19	9	18	8	91	34	13	20	10	10	0.0593
Asafoetida	94	38	24	11	9	12	91	13	11	21	14	31	< 0.001
Fennel seeds	94	34	11	25	12	12	90	27	12	13	17	20	0.1177
Carom seeds	94	31	30	19	8	6	89	32	21	15	13	10	0.374
Black pepper	93	34	28	12	13	6	88	30	10	6	27	18	0.0002
Coriander seeds	91	19	18	25	18	11	87	15	9	30	15	19	0.179
Black Salt	87	24	18	14	18	13	73	20	10	22	9	5	0.0826
Cinnamon	84	1	19	27	15	22	70	4	2	7	23	27	< 0.0001
Dry mango powder	81	6	15	15	21	24	70	2	6	25	17	23	< 0.0001
Fenugreek seeds	79	3	14	16	24	22	69	0	3	19	21	27	0.0317
Mustard Seeds	75	5	7	16	26	21	66	0	2	14	22	32	0.0347
Pomegranate seeds	74	0	12	14	16	32	63	0	1	10	12	46	0.0049
Cloves	71	0	3	6	29	33	63	0	2	6	22	33	0.876
Black cardamom	70	0	3	6	25	36	52	0	1	4	15	30	0.766
Bay Leaf	66	0	5	15	10	36	50	0	1	6	7	36	0.176
Gooseberry powder	60	2	19	10	5	24	50	0	1	1	10	40	< 0.0001
Saffron	55	0	6	3	20	26	39	0	0	6	5	28	0.003
Nigella seeds	42	0	3	4	16	19	28	0	0	1	1	22	0.002
Nutmeg	37	0	2	4	4	27	25	0	0	1	2	25	0.339
Mace	35	0	5	6	4	20	24	0	0	0	7	18	0.014
Dill	33	0	2	4	7	20	23	0	0	0	1	22	0.028
Star Anise	30	0	0	5	5	20	13	0	0	0	1	12	0.174

Statistical Test performed on 5 categories (Daily, twice a week, thrice a week, weekly and occasionally): T Test, statistically significance difference (*p* value < 0.05)

### Mean consumption frequency score among households

The mean consumption frequency score of major spices has been depicted in Table 3. The maximum mean consumption frequency score among urban and rural households was observed for red chilli powder (5.00) and turmeric powder (5.00). The lowest scores were obtained by star anise 0.45 and 0.14 among urban and rural households, respectively. The consumption frequency score revealed that spice consumption was significantly (*p* < 0.01) higher among urban households for cumin seeds, asafoetida, black pepper, gooseberry powder, mustard seeds, pomegranate seeds, dill, nigella, cinnamon and saffron when compared to rural households. The mean frequency score of bay leaf, black salt, black cardamom, carom seeds, green cardamom, fennel seeds, coriander seeds, dry mango powder and clove showed non-significant difference among the adult women of urban and rural households.

**Table 3:** Mean consumption frequency score of major spices consumed by urban and rural households

Spices	Consumption Frequency Score		***p***-value
	Urban (***n =*** 100)	Rural (***n =*** 100)	
**Red chilli powder**	5.00 ± 0.00	5.00 ± 0.00	–
**Turmeric powder**	5.00 ± 0.00	5.00 ± 0.00	–
**Cumin seeds**	4.68 ± 0.78	3.98 ± 1.42	0.00010
**Carom seeds**	3.54 ± 1.45	3.25 ± 1.67	0.24509
**Asafoetida**	3.49 ± 1.62	2.32 ± 1.56	< 0.00001
**Black Pepper**	3.48 ± 1.54	2.80 ± 1.75	0.00397
**Green cardamom**	3.44 ± 1.65	3.12 ± 1.78	0.2877
**Fennel seeds**	3.25 ± 1.60	2.82 ± 1.79	0.10748
**Black Salt**	3.05 ± 1.74	2.29 ± 1.94	0.38208
**Coriander seeds**	2.88 ± 1.53	2.50 ± 1.56	0.05155
**Cinnamon**	2.42 ± 1.74	1.22 ± 1.29	0.00007
**Dry mango powder**	2.01 ± 1.52	1.65 ± 1.37	0.18406
**Fenugreek seeds**	1.87 ± 1.39	1.38 ± 1.18	0.02018
**Mustard seeds**	1.74 ± 1.43	1.26 ± 1.09	0.00402
**Gooseberry powder**	1.55 ± 1.71	0.67 ± 0.77	< 0.00001
**Pomegranate seeds**	1.54 ± 1.33	1.04 ± 0.96	0.00071
**Cloves**	1.21 ± 1.02	1.03 ± 1.00	0.27759
**Bay Leaf**	1.21 ± 1.20	0.76 ± 1.00	0.32275
**Black cardamom**	1.18 ± 1.01	0.76 ± 0.92	0.15865
**Saffron**	0.99 ± 1.15	0.56 ± 0.84	0.00786
**Nigella seeds**	0.75 ± 1.05	0.26 ± 0.48	0.00009
**Mace**	0.66 ± 1.13	0.32 ± 0.60	0.03593
**Nutmeg**	0.55 ± 0.90	0.32 ± 0.56	0.04551
**Dill**	0.54 ± 0.93	0.24 ± 0.45	0.00144
**Star Anise**	0.45 ± 0.80	0.14 ± 0.37	0.03514

Mean ± SD, Mean consumption frequency scores calculated: daily (5), thrice a week (4), twice a week (3), weekly (2) and occasionally (1). Wilcoxon-Mann-Whitney test, statistically significance difference (*p* value < 0.05)

### Usage form of spices by rural and urban households

The form of spices used among households is presented in the Table 4. Results depicted that the spices namely nigella seeds, mace, nutmeg, fennel seeds, star anise and green cardamom were used in their unroasted form by the majority of urban and rural households. A significant difference (*p* < 0.05) was found in using a different form of spices namely green cardamom, black pepper, asafoetida, cinnamon, fenugreek seeds, coriander seeds, bay leaf, clove, carom seeds and black cardamom among urban and rural households. The overall result, it showed that a maximum of 76% of urban and 72% of rural households preferred to use the unroasted form of spices followed by 19% of urban and 21% of rural households using roasted form of spices and remaining using both roasted and unroasted form of spices.

**Table 4:** Usage form of spices by urban and rural households

Spices	Urban			Rural			*p* value
	Unroasted	Roasted	Both	Unroasted	Roasted	Both	
Nigella seeds	97	3	0	89	10	1	0.077
Mace	96	3	1	97	2	1	0.902
Nutmeg	96	4	0	88	11	1	0.099
Fennel seeds	95	5	0	89	10	1	0.239
Star Anise	93	7	0	97	2	1	0.145
Green cardamom	90	10	0	97	2	1	0.037
Black Pepper	87	13	0	66	27	7	0.003
Asafoetida	86	9	5	65	34	1	< 0.001
Pomegranate seeds	84	16	0	92	7	1	0.086
Cinnamon	82	16	2	54	34	12	< 0.001
Fenugreek seeds	79	19	2	65	26	9	0.031
Mustard Seeds	72	27	1	82	17	1	0.232
Coriander seeds	70	29	1	62	25	13	0.004
Bay Leaf	68	32	0	81	18	1	0.048
Cloves	51	40	9	40	36	24	0.015
Carom seeds	41	37	22	58	41	1	< 0.001
Cumin seeds	41	37	22	27	39	34	0.286
Black cardamom	32	35	33	50	37	13	0.001
Total	1360	342	98	1299	378	123	

Statistical Test: T Test, statistically significance difference (*p* value < 0.05)

### Spice intake by adult women among urban and rural households

Spice intake among adult women based on the portion size and quantity of individual spice consumed from all dishes and foods expressed as mean, median, 90th percentile levels and ranges are presented in Table 5. The mean intakes among urban women above 0.18 g/portion size were observed for red chilli powder, turmeric powder, black salt, fennel seeds, cumin seeds, asafoetida, black pepper, green cardamom and for red chilli powder, turmeric powder, cumin seeds, asafoetida, black pepper, carom seeds, fennel seeds, green cardamom, and mustard seeds were observed among rural women. The highest mean intake and range was observed for red chilli powder (3.19 g with range 0.35–5.23 g, respectively) for urban women and (2.41 g with range 0.25–3.75 g, respectively) for rural women. Mean turmeric powder intake among urban and rural households was 2.98 and 2.80 g, respectively. Cinnamon had the lowest mean intake (0.04 g/portion) among urban and 0.2 g/portion for gooseberry powder, 0.03 g/portion for fenugreek seeds and dry pomegranate powder among rural women. A significant difference was obtained between urban and rural households for the intake of red chilli powder, cumin seeds, black pepper, fennel seeds, gooseberry powder and mustard seeds at *p* < 0.01 level of significance. The percent of spice median intakes of spices representing below 1 g were 72 and 89% of urban and rural median intakes, respectively. Out of 18, 10 and 7 spices among urban and rural were having 90th percentile above 1 g, respectively. The highest 90th percentile value was observed for red chilli powder (4.41 g) among urban and turmeric powder (3.23 g) among rural. Overall, an average adult woman consumed 10.04 g of spices per day as compared to 7.68 g per day for rural women indicating higher consumption of spices among urban households.

**Table 5:** Daily intake of spices by adult women of selected urban and rural households

Spice	Mean (g)		***p***-value	Range (g)		Median (g)		90th percentile	
	Urban	Rural		Urban	Rural	Urban	Rural	Urban	Rural
Red chilli powder	3.19 ± 1.26	2.41 ± 0.83	< 0.00001	0.35–5.23	0.25–3.75	2.04	1.20	4.41	2.71
Turmeric powder	2.98 ± 0.90	2.80 ± 1.2	0.09	0.63–4.23	0.28–5.20	1.89	1.55	3.32	3.23
Cumin seeds	0.74 ± 0.79	0.43 ± 0.50	0.0007	0.07–2.71	0.01–1.98	0.83	0.61	2.33	1.30
Black pepper	0.67 ± 1.10	0.27 ± 0.58	0.0008	0.04–4.25	0.03–4.25	1.25	0.19	1.25	2.50
Fennel seeds	0.66 ± 0.99	0.29 ± 0.71	0.0018	0.06–5.00	0.02–5.00	1.25	0.20	1.87	2.50
Green cardamom	0.36 ± 0.38	0.22 ± 0.42	0.0779	0.06–1.63	0.02–2.02	0.44	0.17	1.25	0.94
Black salt	0.25 ± 0.55	0.12 ± 0.31	0.0205	0.25–2.50	0.06–1.25	1.25	0.87	1.37	2.50
Asafoetida	0.24 ± 0.36	0.27 ± 0.44	0.3021	0.18–1.66	0.06–1.96	0.41	0.63	1.50	1.25
Coriander seeds	0.17 ± 0.34	0.11 ± 0.25	0.1017	0.04–1.66	0.01–1.30	0.25	0.12	0.85	0.80
Carom seeds	0.16 ± 0.29	0.20 ± 0.33	0.1905	0.18–1.56	0.06–1.23	0.42	0.44	1.06	0.83
Gooseberry powder	0.16 ± 0.41	0.02 ± 0.08	0.0004	0.02–0.98	0.03–0.71	0.36	0.11	0.66	0.97
Dry pomegranate	0.10 ± 0.34	0.03 ± 0.13	0.2490	0.01–0.90	0.06–0.98	0.12	0.25	0.94	0.86
Mustard seeds	0.08 ± 0.19	0.20 ± 0.36	0.0018	0.11–0.83	0.04–1.85	0.33	0.42	1.24	0.83
Clove	0.07 ± 0.18	0.07 ± 0.15	0.4721	0.03–0.83	0.03–0.78	0.07	0.10	0.63	0.80
Dry mango powder	0.06 ± 0.14	0.08 ± 0.33	0.2490	0.21–0.63	0.20–1.98	0.31	0.60	0.98	0.63
Black cardamom	0.06 ± 0.15	0.07 ± 0.16	0.4170	0.06–0.89	0.01–0.89	0.23	0.08	0.57	0.58
Fenugreek seeds	0.05 ± 0.15	0.03 ± 0.13	0.1264	0.08–0.89	0.04–0.98	0.22	0.06	0.92	0.84
Cinnamon	0.04 ± 0.11	0.06 ± 0.10	0.1391	0.04–0.71	0.04–0.63	0.07	0.10	0.30	0.45
Total	**10.04**	**7.68**							

Mean ± SD, one day intake of spices from vegetable preparations, *paranthas*, *dhal* and other dishes consumed Statistical Test: T Test, statistically significance difference (*p* value < 0.05)

The distribution of the level of spice intake based on portion sizes is depicted in the Table 6. The total number of 1395 and 1107 portion sizes was obtained from the aggregate of all spices consumed from all dishes by adult women among urban and rural households, respectively in 1 day. Maximum number of portion sizes representing more than 8% of total portion sizes belonged to red chilli powder (20.7%), turmeric powder (17.4%), cumin seeds (8.5%), green cardamom (8.4%) and black pepper (8.2%) among urban women and red chilli powder (18.3%), turmeric powder (19%), cumin seeds (8.9%) and black pepper (8%) among rural women. Further, it showed that except for red chilli powder, turmeric powder, cumin seeds, fennel seeds, black pepper and black salt, the rest of the spices showed more than 50% of the portion sizes consumed below 1 g among urban women. While among rural women, except for red chilli powder, rest of the spices showed more than 50% of portion sizes consumed below 1 g. Percent of portion sizes consumed between 1 and 3 g were highest for fennel seeds (70%) followed by turmeric powder (66.7%), cumin seeds (60.1%) and red chilli powder (51.2%) among urban intake. The corresponding highest value was observed for turmeric powder (65.5%) portion sizes consumed between 1 and 3 g. Greater than 3 g, percent portion sizes were observed for red chilli powder (33.9%), turmeric powder (16.9%), black pepper (23.4%) and fennel (3.70%) among urban and red chilli powder (6.7%) and turmeric powder (21.2%) among rural, respectively.

**Table 6:** Distribution of levels of spice intake based on portion size consumed among adult women of urban and rural households

Spice	Urban (*n* = 100)				Rural (*n =* 100)			
	Number of portion sizes	Level of intake (% of portion sizes)			Number of portion sizes	Level of intake (% of portion sizes)		
		< 1.0 g	1.0–3.0 g	> 3.0 g		< 1.0 g	1.0–3.0 g	> 3.0 g
Red chilli powder	289 (20.7)	14.8	51.3	33.9	203 (18.3)	13.3	65.5	21.2
Turmeric powder	243 (17.4)	16.5	66.6	16.9	210 (19.0)	61.4	31.9	6.7
Cumin seeds	119 (8.5)	39.8	60.2	–	99 (8.9)	69.7	30.3	–
Green cardamom	117 (8.4)	89.0	11.0	–	75 (6.8)	78.7	21.3	–
Black pepper	115 (8.2)	30.8	45.8	23.4	89 (8.0)	65.2	34.8	–
Fennel seeds	81 (5.8)	26.3	70.0	3.7	62 (5.6)	74.2	25.8	–
Clove	58 (4.2)	100.0	–	–	40 (3.6)	100.0	–	–
Coriander	57 (4.1)	83.0	17.0	–	43 (3.9)	97.7	2.3	–
Asafoetida	54 (3.9)	81.8	18.2	–	45 (4.1)	80.0	20.0	–
Cinnamon	54 (3.9)	100.0	–	–	42 (3.8)	100.0	–	–
Black cardamom	52 (3.7)	100.0	–	–	39 (3.5)	100.0	–	–
Carom seeds	37 (2.7)	94.6	5.4	–	42 (3.8)	88.1	11.9	–
Black salt	29 (2.1)	44.8	55.2	–	34 (3.1)	61.8	38.2	–
Mustard	22 (1.6)	100.0	–	–	45 (4.1)	80.0	20.0	–
Dry mango powder	19 (1.4)	100.0	–	–	11 (1.0)	54.5	45.5	
Fenugreek	18 (1.3)	100.0	–	–	16 (1.4)	100.0	–	–
Dry pomegranate	16 (1.1)	64.7	35.3	–	7 (0.6)	71.4	28.6	–
Gooseberry powder	15 (1)	62.5	37.5	–	5 (0.5)	70.0	30.0	–
Total	**1395**				**1107**			

Values in parenthesis are percentages, ‘-‘Nil, Coriander, fenugreek and mustard refer to seeds. Portion size of each spice consumed: an aggregate of intakes from vegetable preparations, *paranthas*, *dhal* and other dishes. No. of Portion sizes is no. of portions of each mentioned spice through all dishes consumed in one day by women. Portion size is amount of food that you actually consumed or eat

### Quantity of spice intake per portion size of spice consumed from individual dishes

The spice intake from individual dishes like *dhal*, vegetable and *curry* were evaluated given in the Tables 7, 8 and 9, respectively. *Dhal* (legume preparation), vegetable (dry vegetables) and *curry* (preparations like *rajmah*, *curry*, black *channa*). Maximum number of portion sizes consumed were highest from *dhal* followed by vegetable and *curry* among rural households.

**Table 7:** Quantity of spice intake per portion size of spice consumed from individual dish *dhal* among rural and urban households

***DHAL***											
Spice	No. of portion sizes		Mean (g)			Range (g)		Median (g)		90th percentile (g)	
	URBAN	RURAL	URBAN	RURAL	***p***-value	URBAN	RURAL	URBAN	RURAL	URBAN	RURAL
**Red chilli powder**	62	69	1.88 ± 0.42	1.04 ± 0.64	0.0723	0.41–2.00	0.25–2.75	0.85	0.94	1.45	2.12
**Turmeric powder**	77	82	1.03 ± 0.63	1.44 ± 0.69	0.0009	0.42–3.00	0.41–3.23	1.00	1.25	1.66	2.71
**Mustard seeds**	11	25	0.45 ± 0.06	0.80 ± 0.47	0.0042	0.30–0.50	0.11–1.84	0.50	0.81	0.5	1.59
**Carom seeds**	12	23	0.31 ± 0.14	0.64 ± 0.34	0.0004	0.06–0.42	0.21–1.21	0.42	0.50	0.41	1.14
**Asafoetida**	34	28	0.43 ± 0.14	0.58 ± 0.37	0.0552	0.21–0.83	0.08–1.5	0.42	0.48	0.71	1.25
**Cumin seeds**	74	62	0.66 ± 0.44	0.54 ± 0.53	0.1224	0.09–1.66	0.01–1.98	0.41	0.36	1.25	1.42
**Dry Mango powder**	11	5	0.15 ± 0.23	0.38 ± 0.40	0.18252	0.02–0.75	0.06–0.83	0.06	0.25	0.75	–
**Black pepper**	35	45	0.11 ± 0.11	0.35 ± 0.39	0.0003	0.02–0.63	0.02–1.42	0.10	0.17	0.14	1.11
***Garam masala***	52	47	0.13 ± 0.20	0.89 ± 1.00	0.00006	0.02–1.25	0.06–0.37	0.32	0.28	0.14	0.63

No. of Portion sizes: No. of portions of each mentioned spice through all *dhals* consumed in one day by women. Portion size is amount of food that you actually consumed or eat

Mean ± SD, Statistical Test: T Test, statistically significance difference (*p* value < 0.05)

**Table 8:** Quantity of spice intake per portion size of spice consumed from individual dish *vegetable* among rural and urban households

VEGETABLE											
Spice	No. of portion sizes		Mean (g)			Range (g)		Median (g)		90th percentile (g)	
	URBAN	RURAL	URBAN	RURAL	***p***-value	URBAN	RURAL	URBAN	RURAL	URBAN	RURAL
**Red chilli powder**	69	59	0.86 ± 0.48	0.87 ± 0.45	0.48011	0.26–2.00	0.41–2.50	0.76	0.70	1.67	1.66
**Turmeric powder**	80	70	1.47 ± 0.74	1.35 ± 0.75	0.20710	0.42–5.00	0.20–5.00	1.25	1.20	1.98	1.89
**Mustard seeds**	30	22	0.46 ± 0.13	0.41 ± 0.02	0.08847	0.36–0.76	0.36–0.50	0.41	0.42	0.74	0.43
**Carom seeds**	11	20	0.48 ± 0.25	0.39 ± 0.23	0.17991	0.04–0.99	0.04–0.83	0.43	0.41	0.86	0.83
**Asafoetida**	12	9	0.49 ± 0.14	0.45 ± 0.09	0.23969	0.41–0.79	0.42–0.71	0.42	0.39	0.78	–
**Cumin seeds**	67	56	0.69 ± 0.41	0.75 ± 0.38	0.20995	0.04–1.66	0.06–2.00	0.66	0.62	1.03	1.07
**Coriander seeds**	63	41	0.36 ± 0.56	0.73 ± 0.35	0.00012	0.03–1.35	0.06–2.01	0.12	0.83	1.21	1.00
**Fenugreek seeds**	12	16	0.26 ± 0.35	0.09 ± 0.02	0.06099	0.03–0.98	0.05–0.14	0.07	0.09	0.92	0.14
**Dry Mango powder**	14	7	1.61 ± 0.32	0.13 ± 0.20	< 0.00001	1.14–2.05	0.03–0.80	1.66	0.06	0.62	–
**Black pepper**	34	39	0.29 ± 0.39	0.09 ± 0.02	0.00553	0.04–1.26	0.05–0.14	0.10	0.08	1.06	0.13
***Garam masala***	38	24	0.66 ± 0.33	0.19 ± 0.02	< 0.00001	0.05–0.18	0.09–1.66	0.56	0.63	0.17	1.06

No. of Portion sizes: No. of portions of each mentioned spice through all vegetables consumed in one day by women. Portion size is amount of food that you actually consumed or eat

Mean ± SD, Statistical Test: T Test, statistically significance difference (*p* value < 0.05)

**Table 9:** Quantity of spice intake per portion size of spice consumed from individual dish *curry* among rural and urban households

*CURRY*											
Spice	No. of portion sizes		Mean (g)			Range (g)		Median (g)		90th percentile (g)	
	URBAN	RURAL	URBAN	RURAL	***p-***value	URBAN	RURAL	URBAN	RURAL	URBAN	RURAL
Red chilli powder	39	33	1.04 ± 0.49	0.96 ± 0.70	0.04119	0.42–1.66	0.26–3.33	0.93	0.89	1.76	1.73
Turmeric powder	26	41	1.08 ± 0.75	1.12 ± 0.70	0.44132	0.21–2.50	0.21–2.75	1.25	1.15	1.91	1.89
Carom seeds	42	10	0.31 ± 0.31	0.33 ± 0.29	0.44014	0.41–2.08	0.06–0.72	0.75	0.66	1.16	–
Asafoetida	42	38	0.51 ± 0.26	0.49 ± 0.29	0.46423	0.09–1.74	0.82–1.57	0.83	0.78	0.76	0.96
Cumin seeds	29	36	0.97 ± 0.45	1.11 ± 0.21	0.10178	0.03–1.12	0.09–1.54	0.65	0.53	0.99	1.41
Coriander seeds	17	18	0.89 ± 0.50	0.74 ± 0.38	0.14139	1.00–2.05	0.04–1.06	0.66	0.08	0.80	0.91
Fenugreek seeds	37	30	0.28 ± 0.02	0.21 ± 0.30	0.07512	0.05–0.12	0.02–0.33	0.09	0.07	0.12	0.25
Gooseberrypowder	32	12	1.57 ± 0.31	1.53 ± 0.33	0.41705	0.04–0.11	0.02–0.21	0.16	0.09	0.10	0.11
Black pepper	16	7	0.08 ± 0.02	0.09 ± 0.08	0.28837	0.80–1.25	0.21–1.05	0.76	0.45	1.05	–
*Garam masala*	39	33	1.04 ± 0.03	1.11 ± 0.21	< 0.00001	0.42–1.66	0.26–3.33	1.03	1.59	1.26	1.13

No. of Portion sizes: No. of portions of each mentioned spice through all *curry* dishes consumed in one day by women. Portion size is amount of food that you actually consumed or eat

Mean ± SD, Statistical Test: T Test, statistically significance difference (*p* value < 0.05)

Mean intakes were highest for red chilli powder (1.88 g) from *dhal* among urban households and turmeric powder (1.04) from *dhal* among rural households elicited in Table 7. Common spices added to *dhal* were red chilli powder, turmeric powder, mustard seeds, carom seeds, cumin seeds, *garam masala*, black pepper and asafoetida among urban and rural households. Median intakes above 0.5 g were observed for red chilli powder and turmeric powder among urban households while red chilli powder, turmeric powder and mustard seeds among rural households. The significant difference was obtained for turmeric powder, mustard seeds, carom seeds, black pepper and *garam masala* intake among urban and rural households (*p* < 0.05). While, red chilli powder, asafoetida, cumin seeds and dry mango powder showed a non-significant difference between both households. The highest 90th percentile intake level was observed for turmeric powder 1.66 and 2.71 from *dhal* among urban and rural households, respectively.

Data on the intake of spices from vegetables was depicted in Table 8. The number of portion sizes consumed from vegetables having more than 50 portion sizes were from red chilli powder, turmeric powder and cumin seeds among urban and rural households. Turmeric powder, red chilli powder, mustard seeds, carom seeds, asafoetida, cumin seeds and fenugreek seeds had shown a non-significant difference among the intake of the above-mentioned spices among urban and rural households. Turmeric powder had a median intake of 1.25 and 1.20 g in urban and rural households, respectively. The highest 90th percentile intake level from vegetables among urban and rural households was observed from turmeric powder 1.98 and 1.89 g, respectively.

Another routinely prepared dishes like *rajmah*, black *channa*, *curry*, etc., their spice intake has been presented in Table 9. Mean intake of turmeric powder from *curry* preparation was highest among urban (1.08 g) and rural (1.12 g) households. The non-significant difference was seen among all spices mentioned with exception of red chilli powder and *garam masala*, which showed a significant difference (*p* < 0.05) among urban and rural households. Median values obtained above 0.60 g were observed by red chilli powder, turmeric powder, carom seeds, asafoetida, cumin seeds, coriander seeds, black pepper and *garam masala* among urban households while red chilli powder, turmeric powder, carom seeds, asafoetida and *garam masala* among rural households. The highest 90th percentile of 1.91 and 1.89 g for turmeric powder among urban and rural households, respectively.

Legumes and vegetables *(Dhal*, vegetables, *curry*) are considered as the main contributor of spices apart from other dishes in 1 day intake. A maximum number of portion sizes for red chilli powder and turmeric were from vegetables>*dhal > curry* preparation among urban households. While among rural households the maximum number of portion sizes were from *dhal* > vegetables>*curry* preparation for red chilli powder and turmeric powder.

## Discussion

In Indian cuisine, the usage and consumption of spices are mainly due to their extrinsic flavour. Various methods such as food frequency questionnaires supplemented with weighed records, dietary recall method, and portion size estimation were used in previous studies [7, 8]. The present study tried to assess the spice intake at individual level and comparing the urban and rural adult women intake in the selected households in Ludhiana district of Punjab, India.

The study showed that out of 25 spices studied, most frequently consumed by more than 80 households were 12 and 9 spices among urban and rural households, respectively. Earlier studies in India also showed that consumption frequency of turmeric powder and red chilli powder was higher than most of the other spices which are consistent with the observations in the present study [9]. Consumption frequency of spice varies with regions and areas. Carlsen studied the intake of spices which ranged from 0.80 to 14.7 times per month in European countries which is lower than the consumption frequency of spices observed in the present study [10]. Black salt is frequently used in *lassi* and curds during summers and sprinkled on salads. Carom and cumin seeds are frequently used in vegetable *tadkas* and *paranthas* among both households. In Norway, it was observed that out of 27 different herbs and spices investigated only eight were consumed by one-third of the population which indicated the lower consumption of spices [10]. Ferruci et al. observed regional differences in per capita spice consumption with the Northern and Western regions consuming a lesser number of spices as compared to the Southern regions of India. In Trivandrum, Mumbai and New Delhi, 95% of the total participants (3625) reported consuming 12, 5 and 4 spices, respectively and turmeric powder was the only spice consumed by more than 95% of the population [11].

The usage form of spices in the households also varied indicating that the unroasted form of spices consumed by the majority of urban and rural households. Generally, spices preferred for roasting are black pepper, asafoetida, carom seeds, cumin seeds, fenugreek seeds, mustard seeds, cinnamon, clove and black cardamom during food preparations. Susheela stated that some spices are processed for their microbial stability and removal of extraneous matter and roasting is one of the cooking processes to release characteristic flavour volatiles and undesirable particles [12]. Moreover, there is a possibility that some natural components could be significantly lost during thermal processing because some bioactive compounds are unstable to heat. Thus, heat-processed foods are considered to have lower health-promoting capacity than the fresh one [13]. In order to save time on the roasting process, women preferred to use an unroasted form of spices. In Indian tradition, most of the spices are subjected to roasting before addition to food preparation as roasting enhances the flavour [14].

Spice intake determination is being acknowledged as they possess bioactive compounds and antioxidant properties. Frequency data of intake together with portion size estimations provided a good quantitative estimation of spice intake at individual level rather than opting for the frequency of spice intake alone. The spice intake varies substantially between different countries, states, geographical regions within the same country possessing different dietary habits and patterns and cuisines within the same region. At an individual level, spice intake measurement is a tough and challenging task than estimating the intake of staple foods like cereals and other foods that are consumed in large quantities daily. Sherman and Hash reviewed through traditional cookbooks which revealed the mean number of spices used in 36 countries were 6.5 and 9.3 spices through vegetable and meat-based recipes in India while in European countries mean number of spices ranged from 1.6 to 4.5 and 0.6 to 4.2 spices through meat and vegetable based recipes, respectively [15]. The mean intake of black pepper (0.97 g/portion) and contributing 40% of total portion sizes were from the intake of salads in Hyderabad city [16]. Ferruciet al. stated the median per capita consumption (g/month/person) of chilli powder (35.7%), *garam masala* (33.3%), coriander (33.3%) and turmeric powder (28.6%) in New Delhi. While in Mumbai, chilli powder (58.3%) had the highest median per capita consumption (g/month/person), followed by turmeric powder (21.7%) and cumin seeds (20.0%). Trivandrum was characterized by high consumption (g/month/person) of chilli powder (166.7%) coriander (102.0%) and ginger (373%). Further study revealed that the per capita monthly median intake level of 0.17–4.6 and 3.1 g for cinnamon and cloves, respectively in different regions of India which were found to be higher than stated in the present study [11]. This may be due to different methods of enumerating the spice intake. Per capita intake represents the average consumption of spices at household level while measurements as a portion sizes are based on the actual quantity of spice consumed from the dish in an eating occasion by an individual. In European countries, cinnamon is used in bakery and confectionary items whereas in India it is rarely consumed along with other spices such as cloves and cardamom for rice or sweet preparations. The maximum intake of 0.22 g/kg body weight was noted through rice pudding consumption in Europe. Carlsen et al.assessed the intakes of individual herbs and spices and estimated the median of total spice and herb consumption which was found to be 2.7 g/person/day in Norway and average total intake of herbs and spices was 1.1 g per day with range from 0.19 to 45.0 g from the food frequency questionnaire (FFQ). The total intake of herbs and spices estimated by food frequency questionnaire (FFQ) between men and women showed non-significant results [10]. Intake of total spices from food frequency questionnaire (FFQ) and 3 D weighed food record in Italian diet elicited that FFQ overestimated total spice intake by an average of 3.2 g per day among women subjects [7]. The total intake of seasoning and spice from food frequency questionnaire used in Japan Public Health Center showed that the food frequency questionnaire (FFQ) underestimated the total intake of seasoning and spice i.e. average intake of 5 g/day by more than 85% [8]. Worldwide, the daily consumption of spices varies and often reported as a mixture of common spices used frequently such as red chilli powder, turmeric powder, mustard, black pepper and white pepper. The average daily intake of common spices per person has been estimated as 4.0 g in the USA, 0.5 g in Europe and 1.0 g in New Zealand [17]. In South India, an average daily intake per person of a few spices, 3.08 g for red chilli powder, 0.33 g for black pepper and 0.87 g for turmeric powder were observed [18]. In most households, fennel seeds were consumed as after-meal digestion but in various preparations, their inclusion as a spice was found to be limited. Information regarding the consumption of spices such as cloves, star anise, dill, black cardamom, cinnamon, nutmeg and mace was less documented as compared to red chilli powder and turmeric powder which indicated the low quantity and occasional use of these spices. Studies reported from Norway and Thailand reported the portion size intake of individual spices. The portion eaten per meal of each appropriate spice or herb from the most frequently consumed dishes from 24 Hour recall method showed that various herbs and spices were widely consumed in local North-east Thai diets. Each popular recipe contains at least 3 spices/herbs and total spice/herbs intake was more than 14.7 g/day in Thai diets [19]. Several earlier studies support the observations and indicate the related information made in the present investigation. The average total intake of spices was 3.2 g per day and determined the portion sizes per eating occasion for 5 individual spices (basil dry, basil fresh, cinnamon, oregano and pepper) and 3 spice blends their mean and median ranged from 0.5–1.3 g and 0.2–0.9 g, respectively [10]. The intake of spices in the Hyderabad districtdepicted that intake was as low as 0.01 g for clove and cinnamon and as high for red chilli powder (20 g). Further, high-frequency consumption of spices was indicated by most portion sizes of spices consumed represented by chillies (19%), turmeric powder (18.4%) and cumin (10.4%). Furthermore, this study reported that chillies were consumed between 1 and 5 g by 70% of portion sizes and the remaining 15% were above 5 g [15]. Similar results were observed regarding the spice intake in Thailand and Mexico (5 and 20 g/person/day, respectively) [20]. These intakes were higher as compared to the present study. The regular consumption of turmeric powder and asafoetida in everyday Nepali diets as they consider them as popular household remedies and components of prescriptions used in traditional healing [21]. On the basis of food production statistics, European countries reported the per capita intake of nutmeg and mace to be 0.1 g [22] while in Hyderabad city it was 0.14–0.23 g/portion size found to lowest as compared to other spice consumption [15].

These observations assume relevance for quantifying spice intake since all spices are not consumed on daily basis and intake of individual spices varied with frequency of consumption of dishes. Dishes are considered as main contributor of spices on daily basis. In the present study, intake portion size of spices was estimated not herbs and it depends upon the number of spices used and consumed on a daily basis as monthly intake could not provide relevant results. An average portion size intake of total spices and herbs from habitual dishes which ranged from 4.9 g to 26.1 g in Thailand [18]. Turmeric powder and red chilli powder had maximum number of portion sizes from *curry* which is prepared daily among all households in Southern India. Further, the highest 90th and 97th percentile values for chillies (6.0 and 11.1 g/portion, respectively) were observed from *chutney* and *dhal* [15]. These obtained values were much higher than the results obtained in present study presenting the consumption of spices in Southern India is more as compared to Northern region. Dishes consumed weekly or monthly showed a lower mean total intake of spices than from those consumed daily (10.4 g/day). Conferred that intake of spices differs with a frequency of the type of dish consumed and use of spices which further facilitates in calculating spice intake at the individual level.

## Conclusion

The intake levels of spices were relatively much lower than the other foods. It does not necessarily mean that spices are of little value. It is pertinent to mention that spices do not only add flavor and taste but also have high polyphenolic content and antioxidant potential of vital importance. The frequency of spice intake and portion size at the individual level of adult women in urban and rural households will provide a quantitative estimate of spice intake. The present study conducted in Ludhiana district of Punjab (India) showed the most frequently consumed spices among rural and urban households in Punjabi diets were red chilli powder, turmeric powder, cumin seeds, carom seeds, black pepper, asafoetida, green cardamom, black salt, fennel seeds and coriander seeds. The spice consumption was higher in urban adult women as compared to rural adult women. Further studies are required to explore the importance and intake of spices.

## Data Availability

The authors declared that data supporting the findings of this study are available within the article. Relevant data and its supplementary information are included in the article.
